# Supplementary material for: Sonmat, a citizen-science enabled Kimjang kimchi case study on associations between hand and kimchi microbiota
Source: Microbiol Spectr. 2025 Dec 17;14(2):e00368-25. doi: 10.1128/spectrum.00368-25 (PMC12889100; doi:10.1128/spectrum.00368-25)
Supplement: Supplemental figures — Fig. S1 to S6. [file spectrum.00368-25-s0001.docx]

**Supplementary figures**


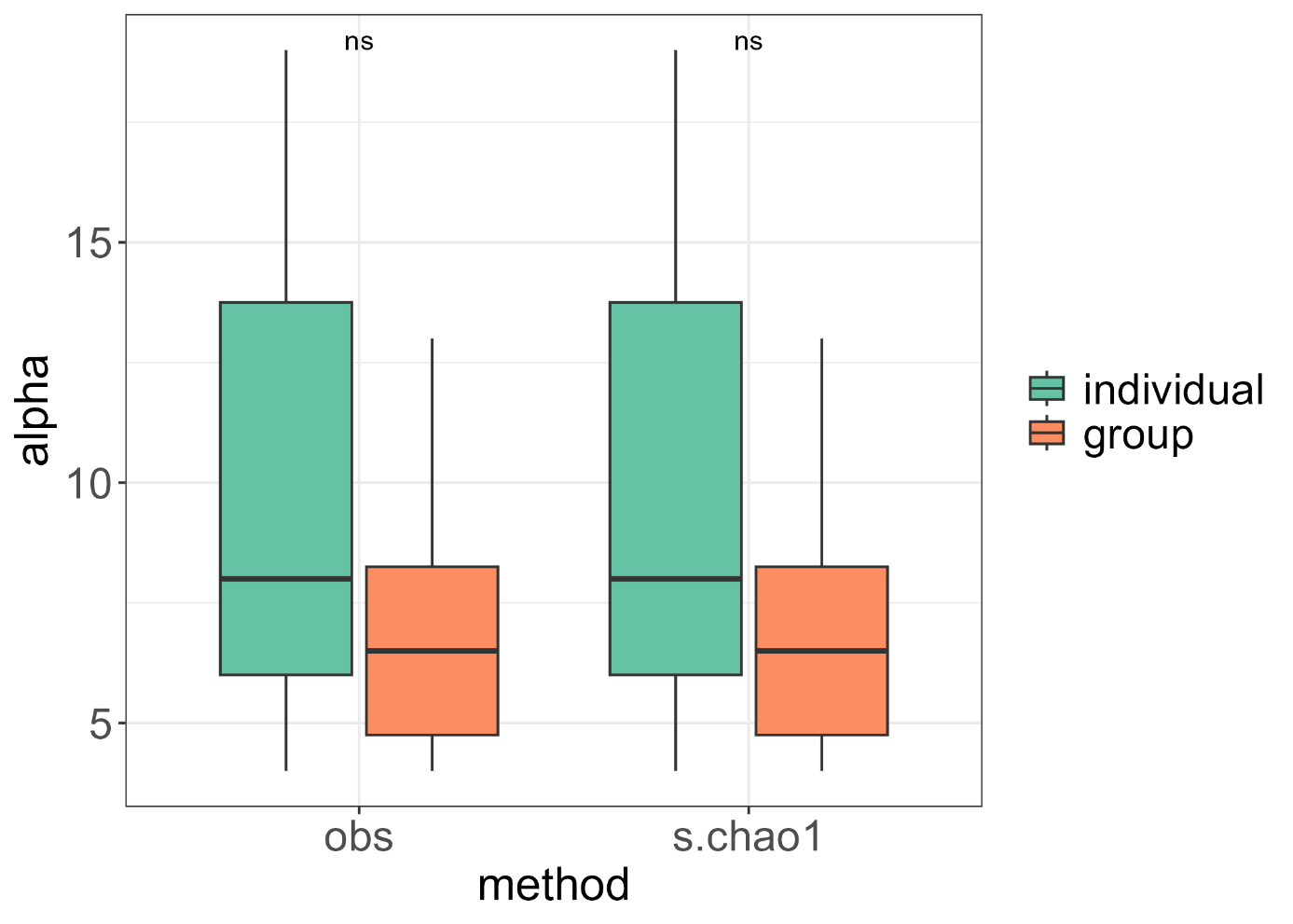


**Figure S1: No significant differences in alpha diversity richness were observed between individually- and group-made kimchi.**  Alpha diversity was calculated with two metrics accounting for richness: observed (obs) and chao1 (s.chao1).


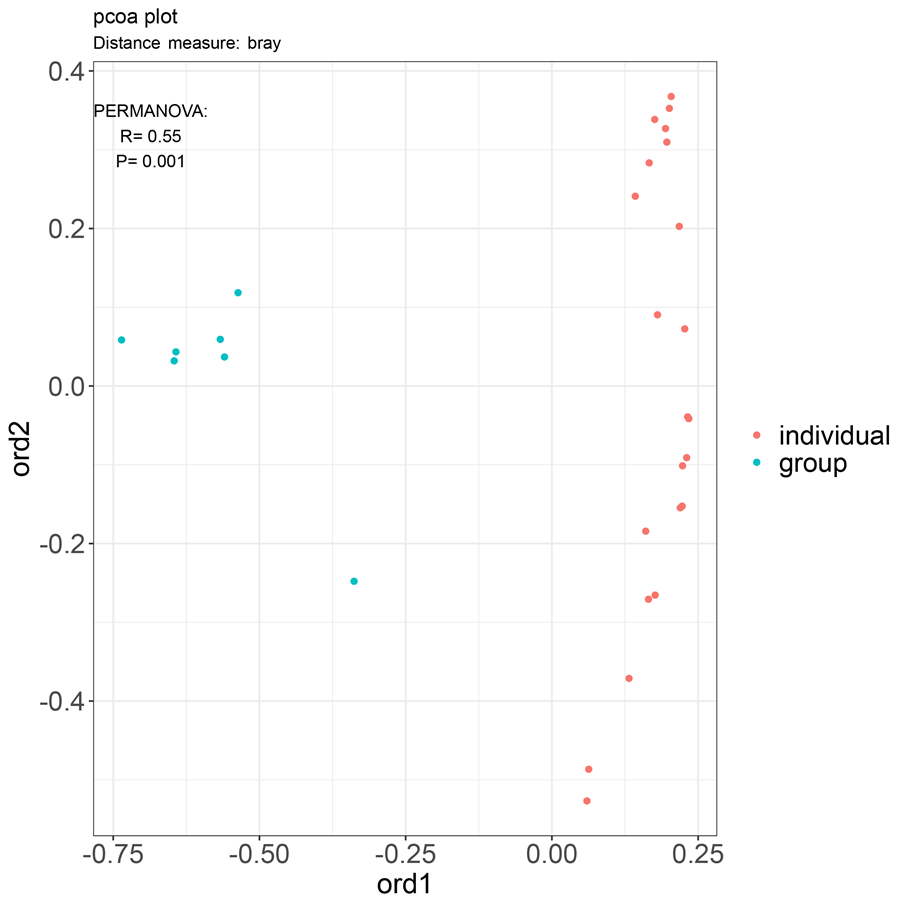


**Figure S2: Significant differences in beta diversity were observed between individually- and group-made kimchi.** Beta diversity was calculated using the Bray-Curtis dissimilarity metric, and significant differences were assessed with PERMANOVA.


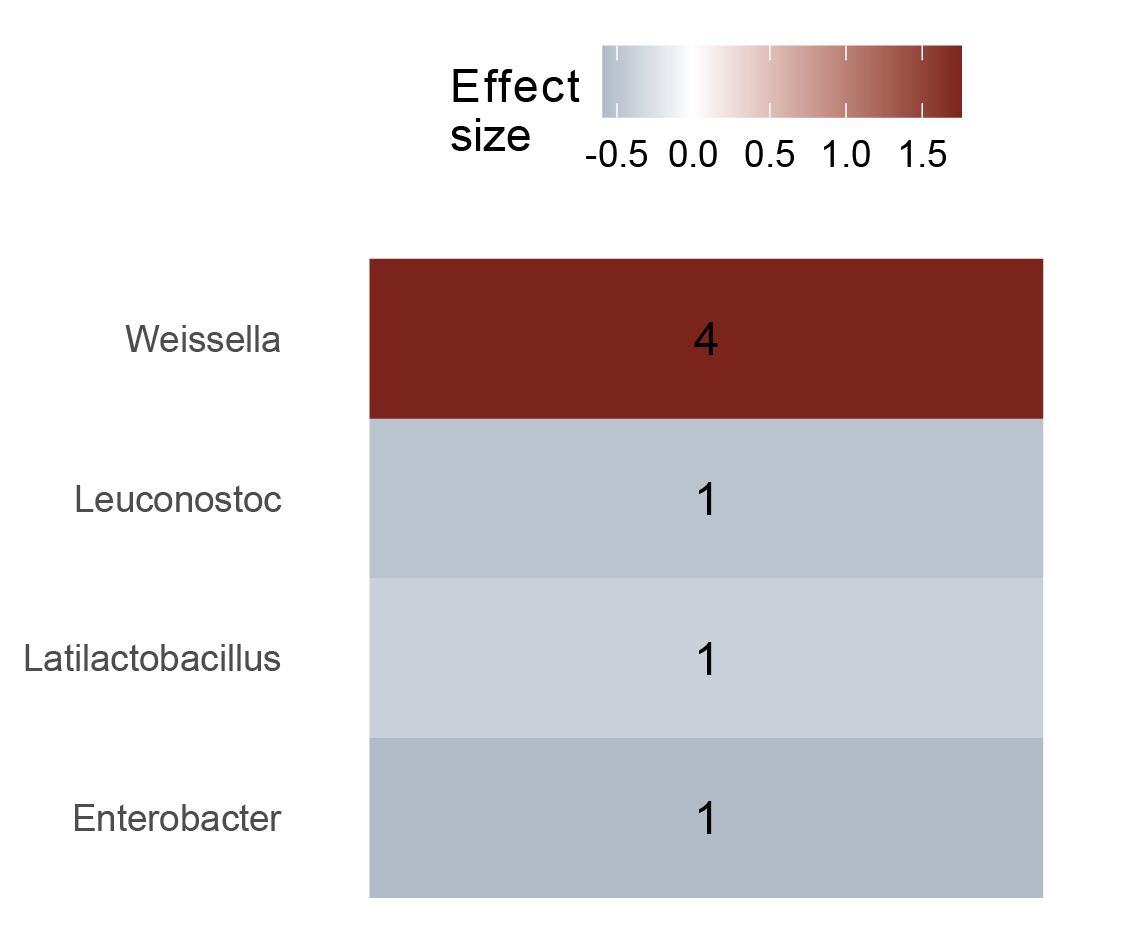


**Figure S3: Differential abundance of microbiota between individually- and group-made kimchi.** Differential abundances of genera with at least 1% mean relative abundance per method (individual or group) were calculated with 4 different tools (ANCOM-BC2, Limma, Deseq2, CLR-Lm) The numbers represent the number of significant tests and boxes were colored based on the effect size of CLR-LM with blue having a higher abundance in kimchi and red a higher abundance on the skin.


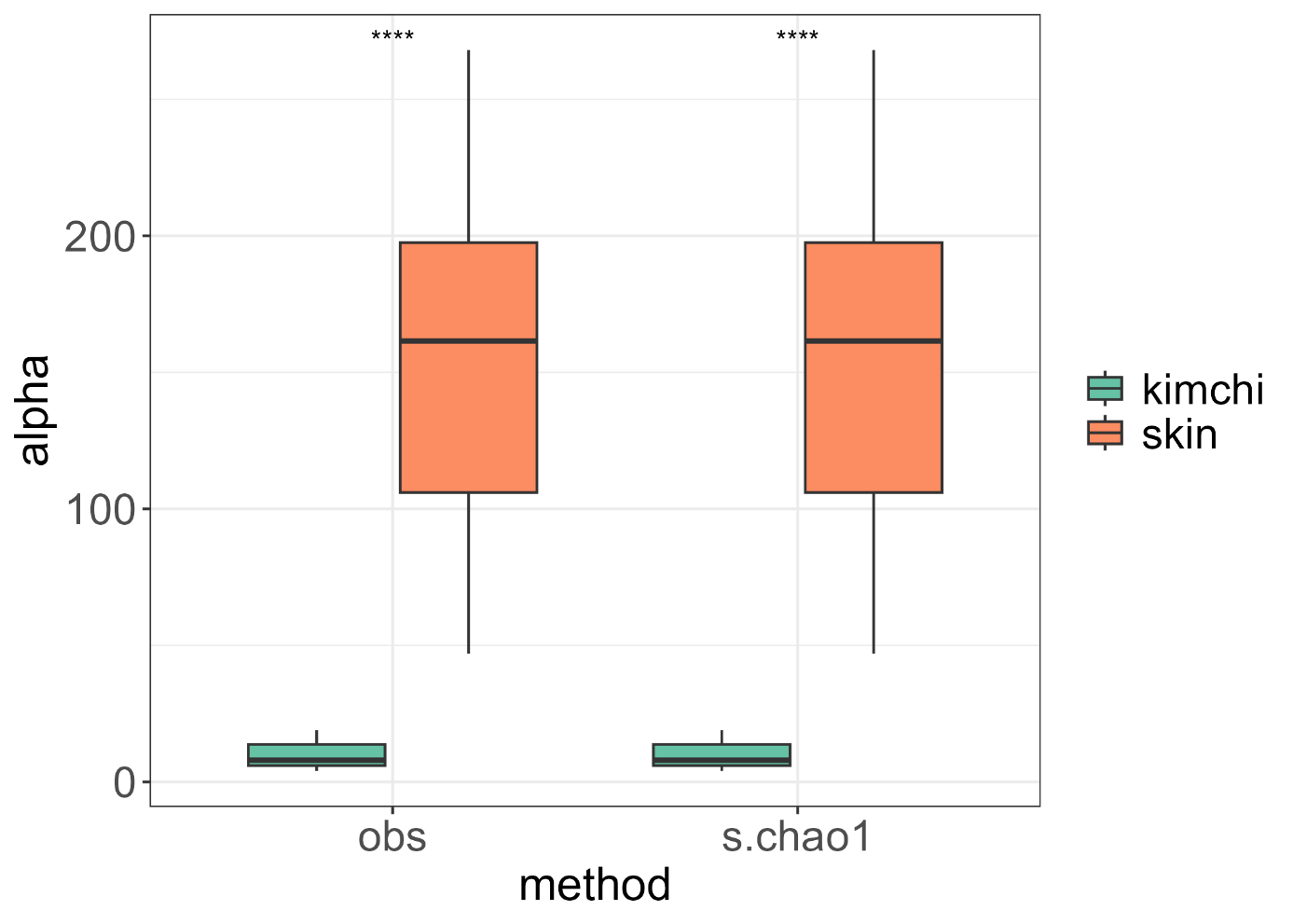


**Figure S4: Significant differences in alpha diversity richness were observed between skin and individually-made kimchi.**  Alpha diversity was calculated with two metrics accounting for richness: observed (obs) and chao1.


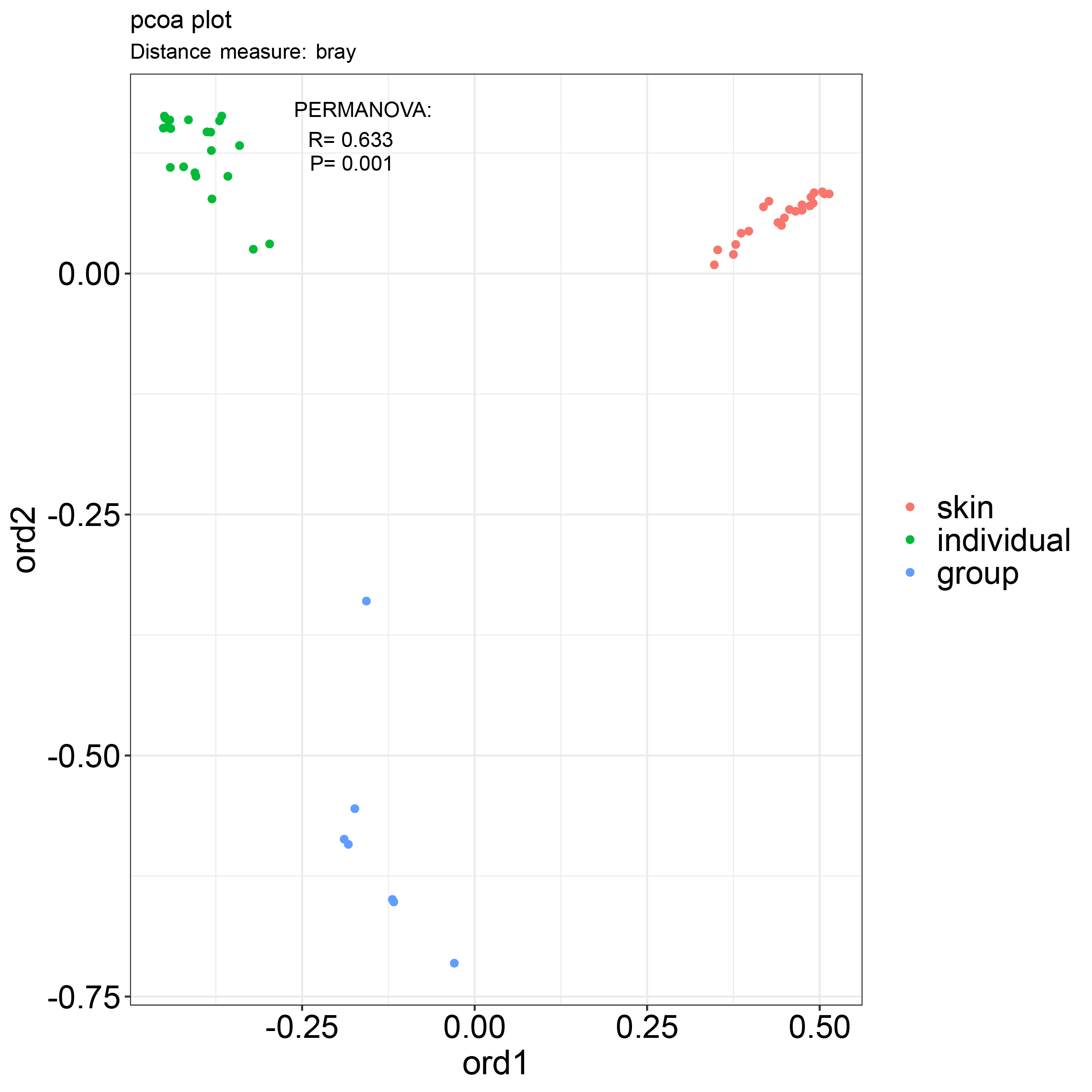


**Figure S5: Significant differences in beta diversity were observed between skin and individually- and group-made kimchi.** Beta diversity was calculated using Bray-Curtis dissimilarity metric and significant differences were assessed with PERMANOVA.


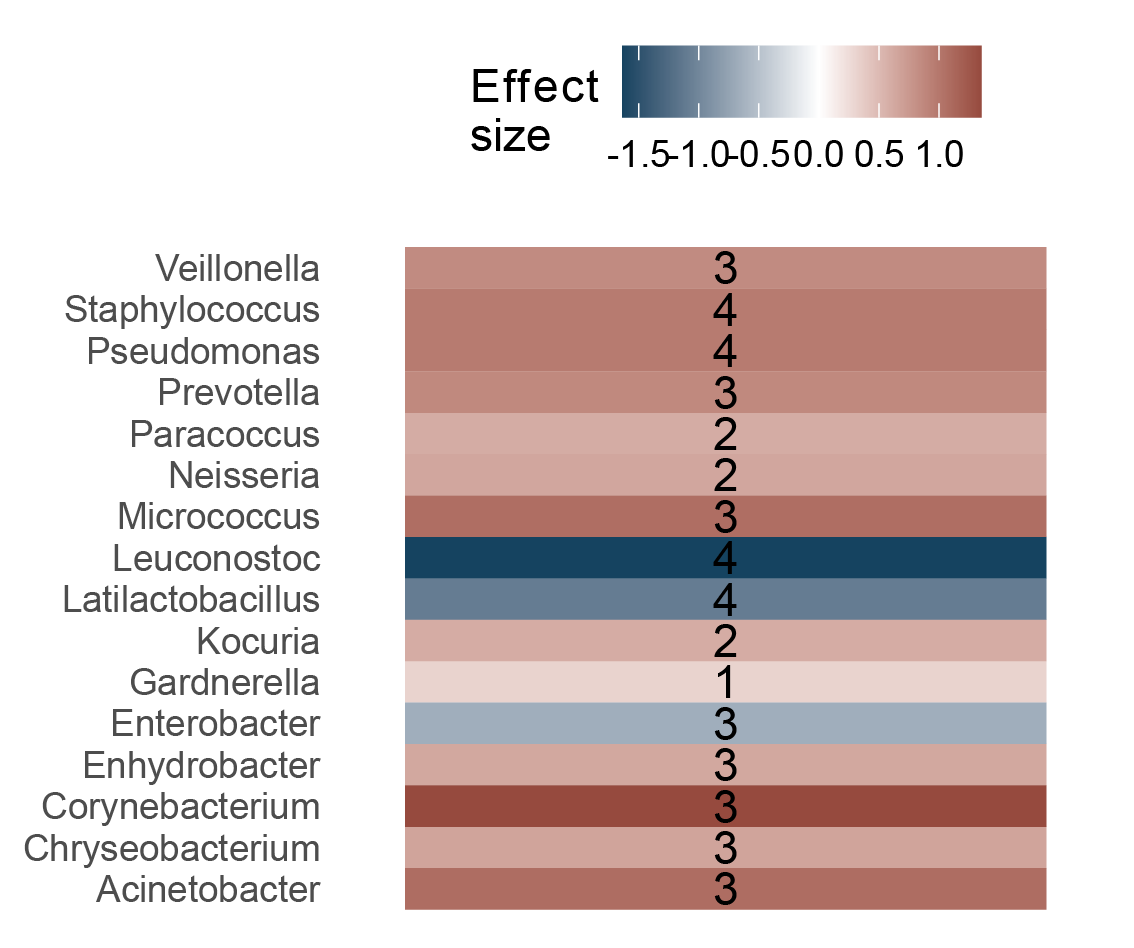


**Figure S6: Differential abundance of microbiota between skin and kimchi microbiome.** Differential abundances of genera with at least 1% mean relative abundance per environment (skin or kimchi) were calculated with 4 different tools (ANCOM-BC2, Limma, Deseq2, CLR-Lm). The numbers represent the number of significant tests and boxes were colored based on the effect size of CLR-LM with blue having a higher abundance in kimchi and red a higher abundance on the skin.
